# Hallmarks of cancer: The insulin-like growth factors perspective

**DOI:** 10.3389/fonc.2022.1055589

**Published:** 2022-11-21

**Authors:** Haim Werner, Derek LeRoith

**Affiliations:** ^1^ Department of Human Molecular Genetics and Biochemistry, Sackler School of Medicine, Tel Aviv University, Tel Aviv, Israel; ^2^ Division of Endocrinology, Diabetes and Bone Diseases, Department of Medicine, Icahn School of Medicine at Mount Sinai, New York, NY, United States

**Keywords:** insulin-like growth factor-1 (IGF1), IGF1 receptor (IGF1R), cancer hallmarks, cell cycle, apoptosis, p53, tumor suppressors

## Abstract

The identification of a series of attributes or *hallmarks* that are shared by virtually all cancer cells constitutes a true milestone in cancer research. The conceptualization of a catalogue of common genetic, molecular, biochemical and cellular events under a unifying *Hallmarks of Cancer* idea had a major impact in oncology. Furthermore, the fact that different types of cancer, ranging from pediatric tumors and leukemias to adult epithelial cancers, share a large number of fundamental traits reflects the universal nature of the biological events involved in oncogenesis. The dissection of a complex disease like cancer into a finite directory of *hallmarks* is of major basic and translational relevance. The role of insulin-like growth factor-1 (IGF1) as a progression/survival factor required for normal cell cycle transition has been firmly established. Similarly well characterized are the biochemical and cellular activities of IGF1 and IGF2 in the chain of events leading from a phenotypically normal cell to a diseased one harboring neoplastic traits, including growth factor independence, loss of cell-cell contact inhibition, chromosomal abnormalities, accumulation of mutations, activation of oncogenes, *etc.* The purpose of the present review is to provide an *in-depth* evaluation of the biology of IGF1 at the light of paradigms that emerge from analysis of cancer hallmarks. Given the fact that the IGF1 axis emerged in recent years as a promising therapeutic target, we believe that a careful exploration of this signaling system might be of critical importance on our ability to design and optimize cancer therapies.

## Introduction to the IGF system

The insulin-like growth factors (IGF1, IGF2) constitute one of the best characterized families of signaling molecules (1–4). The role of the IGFs as mediators of the growth hormone (GH)-stimulated incorporation of sulfate into cartilage was demonstrated more than sixty years ago (5). The specific, GH-activated serum factor that was originally termed *‘sulfation factor*’ and then ‘*somatomedin*’ is now accepted as IGF1. The IGFs developed early in evolution, possibly as regulators of cellular proliferation in relation to nutrient availability (6, 7). Circulating IGF1 levels are dependent on liver production, which is tightly controlled by pituitary-derived GH (8, 9). In addition to its classical endocrine role, many extrahepatic tissues (*e.g*., brain, kidney, stomach, *etc*) produce measurable quantities of IGF1 (10, 11). Locally synthesized IGF1 exhibits tissue-specific paracrine and autocrine activities (12). Both IGF1 and IGF2 activate a common receptor, the IGF1 receptor (IGF1R), which signals mitogenic, antiapoptotic and pro-survival activities (13, 14). The IGF1R is a cell-surface tyrosine kinase receptor coupled to a number of intracellular second messenger pathways, including the *ras-raf*-MAPK and PI3K-AKT signaling cascades (15–17). The IGF1R is vital for cell survival, as illustrated by the lethal phenotype of mice in which the *IGF1R* gene was disrupted by homologous recombination (18–20). IGF2 also interacts with the mitogenic subtype of the insulin receptor (INSR) and thus it is usually more mitogenic than IGF1.

Recent technological developments, including the use of modern genomic and proteomic approaches as well as other high-throughput platforms, are having a huge impact on our understanding of both basic and clinical aspects of the IGF system (21, 22). The unprecedented *gain-of-knowledge* generated by post-genomic technologies is allowing us to analyze physiological and pathological processes at a level of integration that was, until recently, unthinkable (23, 24). Given that the IGF axis and, particularly, the IGF1R emerged in recent years as promising therapeutic targets in oncology, the identification of signaling networks linked to IGF1 action (‘*IGF1 signatures*’) is expected to be of major importance on our ability to optimize interventional tools for the manipulation of this endocrine system (25–31). Furthermore, combined *omics* analyses will most certainly impinge on our capacity to predict responsiveness to selective IGF1R-directed drugs (32, 33).

## Hallmarks of cancer: an emerging unifying concept

The dissection of a complex disease like cancer into a well-defined series of shared genetic, molecular, biochemical and cellular events, or ‘*hallmarks*’, constitutes a true landmark in the history of cancer research. The original catalogue proposed by Hanahan and Weinberg in 2000 included six hallmarks (34). This set of unifying attributes was revised and expanded on a number of occasions to include a number of additional emerging or ‘enabling’ hallmarks (35). For a detailed review of the development of the *Hallmarks of Cancer* concept the reader is referred to the original publications of the authors (36, 37).

As of today, the set of *hallmarks* includes the following ten traits: (1) evasion of growth suppressors (2); avoidance of immune destruction; (3) replicative immortality; (4) promotion of inflammation by tumor; (5) activation of invasion and metastasis; (6) induction or accession to vasculature; (7) genome instability and mutation; (8) resistance to cell death; (9) deregulation of cellular metabolism; and (10) sustained proliferative signaling. Additional emerging hallmarks include: (1) unlocking phenotypic plasticity; (2) non-mutational epigenetic reprogramming; (3) senescence; and (4) polymorphic microbiomes (37).

The purpose of the present review is to provide an *in-depth* analysis of the biology of IGF1 at the light of universal paradigms that emerge from exploration of individual and combined hallmarks. Different aspects of selected hallmarks will be evaluated from the perspective of the IGF1 system. When relevant, the roles of IGF1 and IGF2 in cancer biology will be compared to those of the closely related insulin molecule (38). We believe that this comparison is important from a clinical viewpoint given the vast amount of information linking obesity, hyperinsulinemia and diabetes with cancer initiation and progression (39, 40).

## Sustained proliferative signaling

One of the fundamental attributes of cancer cells involves their proficiency to undergo chronic and, essentially, unlimited proliferation (36). Thus, whereas normal cells exhibit tightly regulated growth signals, transformed cells display a largely deregulated signaling capacity. In most cases, this unrestricted behavior results from the ability of cancer cells to synthesize a variety of growth factor ligands and/or their cognate cell-surface receptors. Growth factor independence may also result from constitutive activation of signaling molecules downstream of the receptors. Regardless of the specific event that is directly responsible for supplying this growth signal, the net outcome is identical, *i.e*., malignantly-transformed cells are able to traverse the cell cycle in the absence of exogenous stimuli in an unopposed manner. In other words, cancer cells exhibit an inherent *sustained proliferative potential*. In this section we will discuss the role of IGF1 in the context of proliferation signaling.

**Figure 1:**
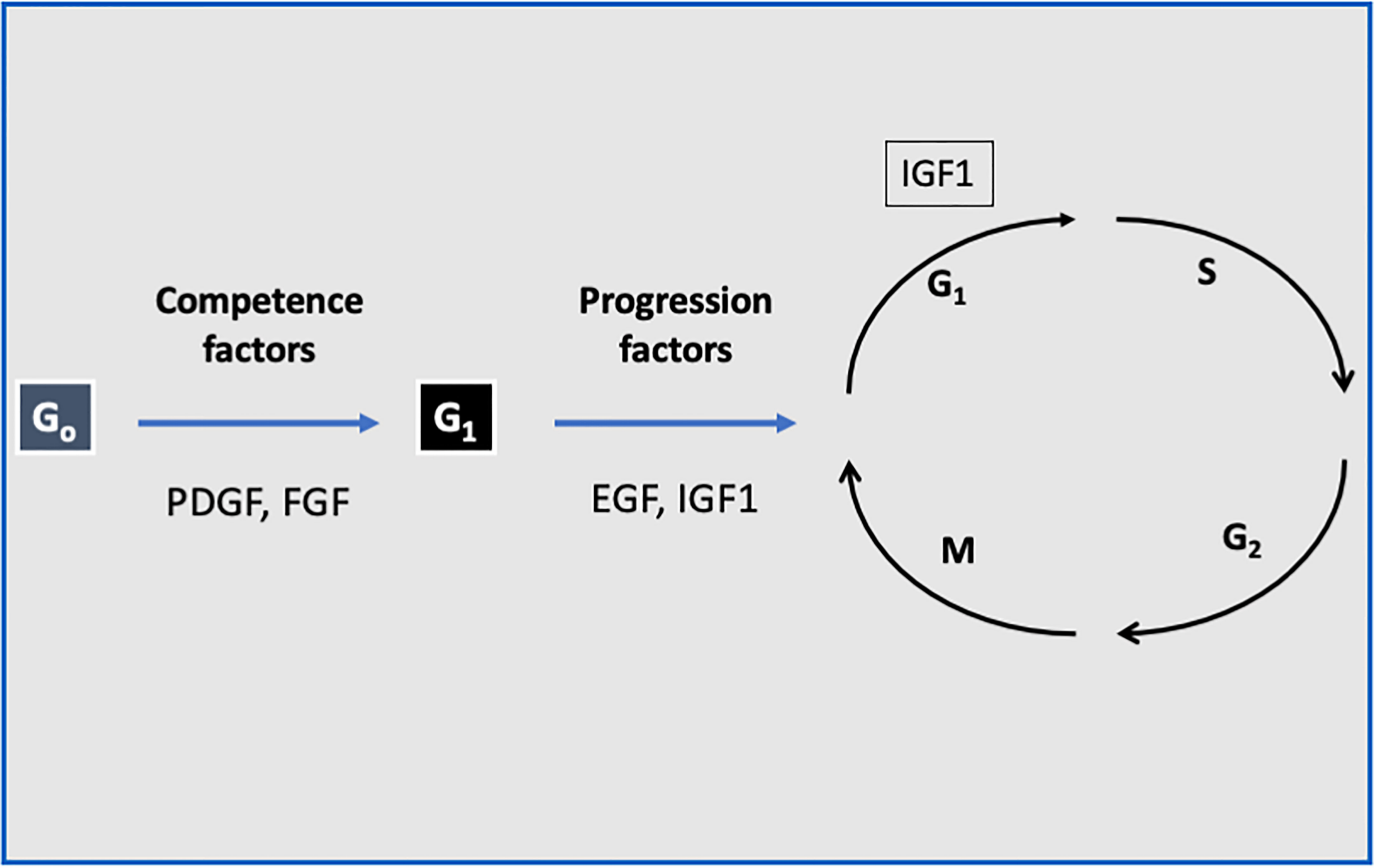
The role of IGF1 as a progression factor. Quiescent cells in G_0_ can be induced to enter G_1_ by competence factors such as PDGF and FGF. Once in G_1,_ the cells require sub-physiological quantities of IGF1 or EGF to evade arrest and to progress through the rest of the cycle. Hence, IGF1 fits the criteria of a progression factor.

The ubiquitous nature of the IGF system has prompted the use of multiple experimental models to study its effects both *in vitro* and *in vivo* (41). At the cellular level, IGF1 stimulates proliferation and inhibits death in a wide variety of cell types (42). IGF1 stimulates a mitogenic response in primary cultures of cells from various origins as well as in cancer cell lines. Of importance, IGF1 has a fundamental role in stem cells biology (43, 44). IGF1 fits the criteria of a *progression factor*, *i.e*., a molecule that is expressly required to traverse the cell cycle (45, 46). Quiescent cells in G_0_ can be induced to enter G_1_ by competence factors (*e.g*., PDGF, FGF). Once in G_1,_ the cells require sub-physiological quantities of IGF1 to evade arrest and to progress through the rest of the cycle (47) (**Figure 1**). IGF1 can also induce differentiation (48), while antisense oligonucleotides against the *IGF1* gene blocked this effect (49).

IGF1 induces a variety of cell- and organ-specific functions, ranging from regulation of hormone synthesis and secretion (50), chemo-attractant migration (51) and neuromodulation (52). IGF1 also participates in cell recognition by the immune system. Thus, glioblastoma cells in which IGF1 expression was disrupted by antisense oligonucleotides generated a strong host response and didn’t form tumors when injected into syngeneic mice (53). In the central nervous system, the *IGF1* gene is widely expressed and promotes proliferation, survival and differentiation of neuronal and non-neuronal cells. In rat brain, distinct regions (*e.g*., cerebellar neurons, retina, sensory and trigeminal ganglia) express high IGF1 mRNA levels during embryonic development while other regions (*e.g*., midbrain, cerebral cortex, hippocampus) expresses the gene mainly during postnatal growth (54). Up-regulation of IGF1 in the central nervous system is observed 1-7 days after a variety of insults, including hypoxia-ischemia (55), brain contusion (56) and penetrating brain trauma (57). Of notice, IGF1 has been identified as a neurotrophic factor, rescuing neurons from apoptosis (58) and enhancing neuronal growth and myelination (59). The role of IGF1 as an anti-apoptotic factor will be discussed below.

Finally, it is important in this context to discuss the mechanisms associated with IGF1 action (12). Ligand binding to the IGF1R extracellular α-subunits results in conformational changes that induce autophosphorylation of tyrosine residues within the mainly intracellular β-subunits (13). Autophosphorylation stimulates the receptor tyrosine kinase activity and leads to phosphorylation of additional substrates. A number of SH_2_ domain-containing proteins, or ‘*docking proteins’*, bind to specific phospho-tyrosine residues in the C-terminal portion of the β-subunit (17). The insulin receptor substrate (IRS) family of proteins and Shc are the best characterized docking proteins. Thus, enzymatic activation of the IGF1R tyrosine kinase domain results in stimulation of an array of intracellular cascades, including the *ras-raf*-MAPK and PI3K-AKT pathways. Classically, IGF1-induced mitogenesis was primarily attributed to the *ras-raf*-MAPK pathway whereas the anti-apoptotic effect of IGF1 was thought to be mediated by the PI3K-AKT pathway. Today, it is clear that the situation is, in fact, much more complex. Whereas in the past IGF1R pathways (like growth factor cascades in general) were depicted as linear tracts, it has become increasingly evident that there is a cross-talk between IGF1R and additional cell-surface receptors, including G-proteins, integrins, and others (12).

## Insensitivity to antigrowth signals

The capacity of normal adult cells to remain in a post-mitotic, terminally differentiated state is dictated by their ability to respond to a series of secreted, cellular or extracellular growth inhibitors. These antiproliferative signals operate to keep the cells out of the cell cycle and in a quiescent state. One of the prototypical cancer hallmarks refers to the acquired faculty of transformed cells to evade these antigrowth signals (34, 37). As a result, cells might regain a previously repressed mitogenic potential that would allow them to re-enter the cell cycle.

The E2F family of transcription factors plays a key role in regulating the expression of genes involved in the G_1_/S transition and DNA synthesis (60–62). The retinoblastoma (Rb) and E2F proteins form a complex (Rb-E2F) that undergoes dissociation upon phosphorylation of Rb, with ensuing activation of E2F-dependent transcription and cell cycle progression (63, 64). E2F binds to DNA and regulates the expression of genes involved in cell cycle progression. Microarray analyses of E2F1-induced genes revealed that genes associated with proliferation as well as apoptosis are usually upregulated by E2F1 (65–67). Using transient transfection assays we have demonstrated that E2F1 is a potent inducer of *IGF1R* gene expression in prostate cancer cells (68). Augmented IGF1R levels correlated with elevated phospho-IGF1R values, suggesting activation of the IGF1R signaling pathway. Deletion analysis indicated that the ability of E2F1 to stimulate *IGF1R* promoter activity correlated with the number of E2F1 sites present in the various constructs, suggesting a dose-dependent effect of E2F1 binding on *IGF1R* gene expression. In addition, *in vivo* analysis of promoter occupancy by chromatin immunoprecipitation assays revealed that E2F1 was specifically recruited to the *IGF1R* promoter. Combined, data indicate that transcription factor E2F1 is an important regulator of the *IGF1R* gene. Elevation in IGF1R levels may contribute to the proliferative effects associated with initiation of prostate (and other) cancer (30, 69).

**Figure 2:**
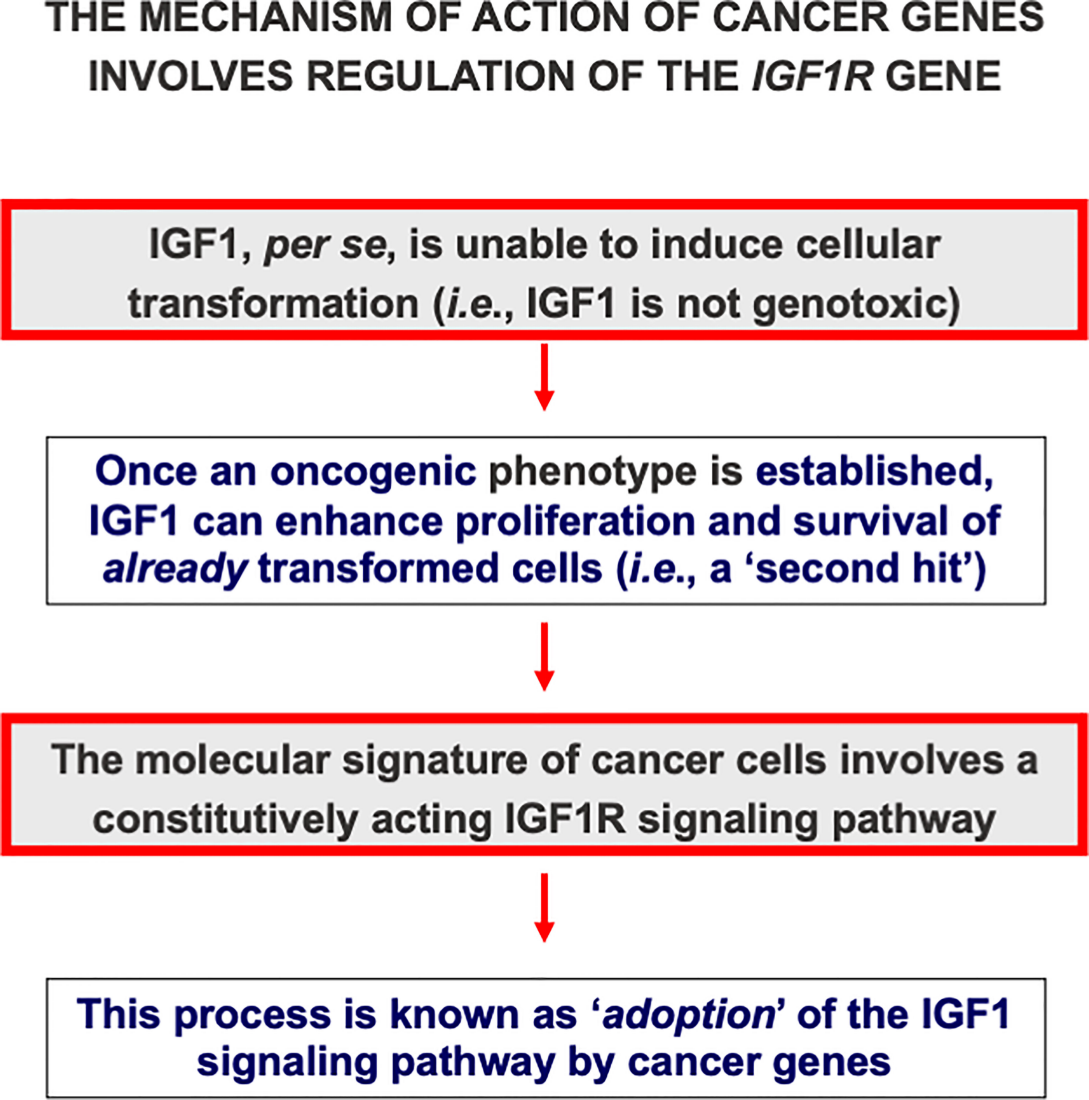
Cancer genes adopt the IGF1 signaling pathway. IGF1 is regarded as a non-genotoxic growth factor, *i.e.*, it is unable, in itself, to induce mutations or transformation. However, once an oncogenic event has occurred, IGF1 can enhance proliferation and survival of already transformed cells. In the context of the ‘*two hit hypothesis’*, IGF1 action is regarded as a second, or permissive, hit. Multiple cancer genes, including oncogenes and anti-oncogenes, adopt the IGF1 signaling pathway. In agreement with this notion, cells devoid of the IGF1R usually do not undergo transformation.

Classically, *loss-of-function* mutations of tumor suppressor genes or *gain-of-function* mutations of oncogenes are regarded as critical events in cancer development. In terms of the ‘*two-hit hypothesis*’ these events fit the criteria of a first, *i.e*. oncogenic, event. A mechanism of action that is shared by multiple oncogenes involves the transactivation of different growth factors or growth factor receptors, including IGF1R. Activation of IGF1 axis components suits the definition of a second, *i.e*. permissive, hit. This mode of action is commonly referred to as ‘*adoption*’ of the IGF1R signaling pathway by oncogenes (**Figure 2**). For example, pp60^src^, the protein encoded by the *src* oncogene of Rous sarcoma virus stimulates the constitutive phosphorylation of the IGF1R tyrosine kinase domain (70). Hence, pp60^src^ alters growth regulation by rendering the cells constitutively subject to a mitogenic signal. Other oncogenes, including c-myb, can *trans*activate the *IGF1R* promoter, with enhanced *IGF1R* gene transcription and biosynthesis (71). In summary, cellular and viral oncogenes require an intact, activated IGF1R signaling pathway in order to elicit their transforming activities (69, 72).

## Evasion of apoptosis

A quintessential feature of cancer cells involves the acquisition of molecular and genetic means that will allow them *not-to-die*. The ability of transformed cells to endure is dictated not only by their proliferative potential but also by their capacity to oppose death, particularly apoptosis. As stated by Hanahan and Weinberg in their original report, acquired resistance towards apoptosis is a fundamental hallmark of, most probably, all types of cancer (34). Classically, programmed cell death has been regarded as an ‘*altruistic*’ mechanism that offers protection to the entire organism by engaging in a self-annihilation program (73). Extensive research over more than fifty years has identified complex biochemical and molecular strategies that are acquired by cancer cells and that are directly responsible for evasion of apoptosis.

Seminal studies from the laboratory of Renato Baserga generated early evidence that the IGF1R exhibits a very potent anti-apoptotic activity in comparison to most other growth factor receptors (74–78). Using a series of IGF1R mutants, O’Connor et al. demonstrated that the domains of the IGF1R required for its anti-apoptotic function are distinct from those required for proliferation or transformation (79). Of notice, IGF1 inhibition of apoptosis occurs in the absence of protein synthesis and, therefore, does not require immediate gene expression (80). Finally, protection from apoptosis is evident in the post-commitment (*i.e*., mitogen-independent) S-G_2_-M phases of the cell cycle.

The inherent anti-apoptotic activity of the IGF1R confers upon receptor-expressing cells enhanced survivability, a fundamental property of cancer cells. Consistent with this notion, fibroblasts (R-) derived from *IGF1R* ‘knock-out’ embryos (the total deficiency of IGF1R is a lethal condition) do not undergo malignant transformation when exposed to oncogenes (81, 82). Reintroduction of a functional receptor renders R- cells susceptible to the transforming activities of these oncogenes. Certain exceptions to this general paradigm have been reported. For instance, transfection of the GTPase-deficient mutant G_α13_ resulted in transformation of R^-^ cells. These results indicate that G_α13_ can induce cellular transformation through pathways independent of IGF1R (83). Taken together, studies are in agreement with the notion that IGF1R expression and/or activation are fundamental pre-requisites for cancer development (8, 20) (**Table 1**). It is important to understand, however, that IGF1, *per se*, is neither genotoxic nor oncogenic. In other words, even supra-pharmacological doses of the hormone cannot induce malignant transformation.

**Table 1:** IGF1R gene expression in human cancers.

Cancer	Reference
Ovary	Bruchim et al. (84)
Endometrium	Bruchim et al. (85)
Breast	Yerushalmi et al. (86)
Thyroid	Wang et al. (87)
Colon	Codony-Servat et al. (88)
Lung	Macaulay et al. (89)
Stomach	Lowe et al. (90)
Kidney	Chin and Bondy (91)
Ewing sarcoma	Mancarella and Scotlandi (27)
Brain	García-Segura et al. (54)

Enhanced IGF1R gene expression constitutes a common feature of most human cancers. IGF1Rs in tumors have been characterized using competitive binding assays, affinity cross-linking, Northern blots, RNase protection assays, RT-PCR, or a combination of them. The table represents a partial list of tumors in which IGF1R is highly expressed.

The key role of the mitochondria in the regulation of biochemical and molecular events associated with apoptosis have been described by Green and Reed (92). Recent genomic analyses identified the thioredoxin interacting protein (TXNIP) as a novel target for IGF1 and insulin action (93). TXNIP is a mitochondrial protein that belongs to the α-arrestin family and plays a key role in redox regulation (94–98). TXNIP binds to the catalytic active center of reduced thioredoxin and inhibits its expression and activity (99). TXNIP is highly expressed in lymphoblastoid cells derived from Laron syndrome patients, a type of congenital IGF1 deficiency. The role of the *TXNIP* gene as a downstream target for negative regulation by IGF1 was confirmed by studies showing that IGF1 (or insulin) treatment led to marked reductions in TXNIP levels in cultured cells. Furthermore, transfection studies revealed that the effect of IGF1 on *TXNIP* gene expression was mediated at the level of transcription (93). We envision a scenario in which IGF1 could inhibit apoptosis by down-regulating TXNIP at the transcriptional level. Independent of redox regulation, TXNIP also functions as a regulator of glucose metabolism (100) and its levels are increased in diabetes (101).

## Genome instability and mutation

Genome instability and mutation were categorized as an enabling cancer hallmark that serves as a pre-requisite for some (possibly most) of the previously described hallmarks (36). The biological rationale for this trait relies on the recognition that specific mutations might confer upon certain cell populations distinctive advantages that could, eventually, facilitate their selective expansion and dominance.

Tumor suppressor p53 is a transcription factor that typically accumulates in the cell in response to DNA damage (102). Mutation of the *p53* gene is the most common event in human cancer (103, 104). When hyperphosphorylated, p53 arrests cell cycle progression at the G_1_ phase. The p53 pathway is activated in response to different stress signals, including DNA damage and telomere shortening, hypoxia, heat and cold shock, inflammation and activation of oncogenes by mutations (105, 106). These various strains bear the potential to decrease the fidelity of cell cycle progression and DNA replication, thus leading to increased mutation rates (107, 108). As alluded to above, accumulation of mutations constitutes an early event in malignant transformation. p53-mediated cell cycle arrest enables damaged DNA to be repaired before the replicative phase of the cell cycle (104, 109). Alternatively, p53 can elicit an apoptotic program. Of relevance, evidence gathered in recent years indicate that, in addition to its well established ability to control cell cycle progression, p53 activation has a major impact on metabolic processes, including glucose transport (110) and obesity (111).

Extensive molecular and genetic analyses revealed that the mechanism of action of wild-type p53 involves transcriptional suppression of the *IGF1R* gene (112). *Gain-of-function*, or *loss-of-function*, mutations of *p53* in tumor cells seem to disrupt its inhibitory activity, generating oncogenic molecules capable of transactivating the *IGF1R* gene. Because p53 is a potent inducer of apoptosis, we assume that the effect of this molecule on apoptosis is mediated, at least in part, *via* suppression of the *IGF1R* promoter. Lack of *IGF1R* inhibition by mutant p53 molecules may help expand cancer cell populations that are otherwise destined to die (113). The ubiquitin ligase Mdm2 is of major importance in regulation of p53 activity (114) and IGF1 was shown to induce p53 degradation in an Mdm2-dependent manner (115). Girnita et al. have shown that Mdm2 physically associates with IGF1R and causes its ubiquitination and degradation (116). Mdm2 serves as a ligase in ubiquitination of IGF1R and thereby causes its degradation by the proteasome system. Consequently, by sequestering Mdm2 in the cell nuclei, the level of p53 may indirectly influence the expression of *IGF1R*. This function of Mdm2 and p53 constitutes a potential mechanism for the regulation of IGF1R and cell growth. As an operational outcome to these studies, IGF1R was identified as a molecular determinant for response to p53 reactivation therapy in conjunctival melanoma (117).

The characterization of the mechanisms responsible for regulation of the IGF1 pathway by p53, as described above, led us to formulate an hypothesis aimed at offering a generalized paradigm for regulation of IGF1R expression by different tumor suppressors (69, 113, 118). While tumor suppressors might differ in their organ-specific expression, mechanisms of activation, type of tumors involved and other parameters, they share the IGF1R pathway as a common response path. This unifying model may explain the involvement of the IGF1 axis in genome instability and mutations.

The breast and ovarian cancer susceptibility gene (BRCA1) is a transcription factor involved in DNA damage repair, cell growth and apoptosis (119, 120). Mutations of the *BRCA1* gene are detected in a significant proportion of families with inherited breast and/or ovarian cancer (121, 122). Transfection of a BRCA1 expression vector in breast cancer cells led to a marked reduction in endogenous IGF1R levels and promoter activity (123–125). In contrast, a mutant *BRCA1* gene encoding a truncated version of the molecule (del185AG, a mutation with a high incidence among Ashkenazi Jews) had no effect on *IGF1R* expression. Hence, activation of BRCA1 in response to DNA damage, oxidative stress, or other cellular insults, may lead to a reduction in IGF1R levels and IGF1 action. Suppression of the IGF1 pathway is expected to prevent from cells from engaging in mitosis.

## Sustained angiogenesis

The ability to grow new blood vessels, or angiogenesis, represents an important capability that neoplasms develop to increase in size and, ultimately, to endure (126). This feature is critically required by the proliferating cell in order to obtain nutrients and oxygen (127). The labeling of sustained angiogenesis as a hallmark of cancer reflects the universal nature of this trait (34). The process of angiogenesis is tightly regulated by secreted growth factors and their receptors as well as by cellular and extracellular adhesion molecules (*i.e*., integrins, cadherin, *etc*). The single most important protein that epitomizes the angiogenic process is vascular endothelial growth factor (VEGF) (128, 129).

The physiological role of VEGF is to induce the formation of new blood vessels during embryonic development and to restore injured vessels (130). Members of the VEGF family (VEGF-A, -B, -C, -D, and placenta growth factor, PGF) are produced by many types of cancer, being the expression of VEGF-A usually correlated with metastatic potential. VEGF-A enhances migration and mitosis of endothelial cells, stimulates matrix metalloproteinase activity and augments integrin αvβ3 activity (131). The *VEGF-A* gene is regarded as an hypoxia-inducible gene, being its transcription regulated by hypoxia-inducible factor-α (HIF-α) (132). In hypoxia (low oxygen pressure), the absence of oxygen-dependent hydroxylation of HIF-α prolines allows HIF-α subunits to accumulate, dimerize and translocate to the nucleus, triggering transcription of *VEGF-A* and other hypoxia-inducible genes (133). The IGF axis enhances the hypoxic response by activation of Akt signaling, leading to stabilization of HIF-α and upregulation of VEGF-A (134, 135).

The von Hippel-Lindau gene product (pVHL, the substrate recognition component of an E3 ubiquitin ligase complex) has an important role in the oxygen-dependent proteolysis of HIF-α (136). Inactivation of VHL in clear cell renal cell cancer (CC-RCC) allows normoxic accumulation of HIF-α subunits, leading to constitutive expression of the angiogenic *VEGF-A* gene. We have previously identified a new hypoxia-independent role for VHL in suppressing *IGF1R* transcription and mRNA stability (137). IGF1R levels were higher in CC-RCC cells harboring a mutant inactive VHL than in isogenic cells expressing a wild-type VHL. Hence, mutant VHL leads to IGF1R upregulation, an event typically associated with renal tumorigenesis. Taken together, data indicate a functional interplay between the IGF1R and VEGF signaling pathways (138). Dysregulation of specific components in these paths might lead to sustained angiogenesis, an important hallmark of cancer.

## Tissue invasion and metastasis

The ability of tumor cells to invade adjacent tissues and to colonize remote sites in the body, (*i.e*., metastasis), is responsible for the vast majority of deaths from cancer (139). This cancer hallmark depends, to a large extent, on hallmarks described above (34). The processes of invasion and metastasis have been extensively investigated over the years from both basic and translational angles. Research led to the identification of molecules and signaling pathways that are directly involved in pathological changes at the interface between malignant cells and the microenvironment. Most of the clinically-relevant changes can be ascribed to proteins involved in cell-cell adhesion, including integrin, cadherin and others (140–143).

Accumulating experimental and epidemiological evidence provide support to the idea that obesity is an important risk factor for cancer (39, 144, 145). A number of mechanisms by which obesity contributes to tumor progression have been described. Obesity is associated with systemic hyperinsulinemia as well as differences in circulating IGFs, adipokines and cytokines (146). The contribution of these molecules to proliferative and cell-survival events has been well documented (147). The increase in adipose tissue in the tumor microenvironment is a source of lipids that can be used by tumors for metabolism and as structural and signaling molecules. In this context, cholesterol was shown to affect gene expression of the jun family in colon cancer cells, leading to potentially pathogenic signaling events (148).

Obesity also leads to changes in the extracellular matrix, adipose stromal cells and immune cells, creating a cancer-permissive microenvironment. The reader is referred to a recent comprehensive review article by Vella et al. that provides a thorough analysis of the interplay between estrogens, insulin/IGFs and their receptors, and stroma (149). Regarding the mechanisms of action responsible for these interactions, the estrogen receptor-α (ERα) has been identified as a potent transactivator of the *IGF1R* gene (30, 150, 151). Furthermore, GC-rich sequences in the proximal *IGF1R* promoter region were required for this effect. Impaired interactions between ERα and zinc-finger proteins may lead to aberrant IGF1R expression in breast cancer cells. Dysregulated expression and availability of IGFs are regarded as key regulators of metastasis (152). Recent genomic analyses identified the nephronectin (NPNT) gene as a downstream target for IGF1 action (153). NPNT is an intracellular and secreted extracellular matrix protein with important roles in kidney development (154, 155). NPNT interacts with α8β1 integrin through its central linker segment. NPNT expression correlated with poor prognosis in breast cancer and was shown to promote metastasis *via* its integrin-binding motif (156, 157). Our analyses identified NPNT as the top down-regulated gene in Laron syndrome cells, a condition associated with diminished IGF1 levels (158).

Finally, constitutive activation of the IGF1R was shown to affect lineage differentiation during mammary tumorigenesis (159). Constitutive IGF1R activation promoted tumors with mixed histology and multiple cell lineages. In these tumors, IGF1R expanded the luminal-progenitor population while influencing myoepithelial differentiation. Combined, the capacity to affect lineage differentiation may promote heterogeneous mammary tumors and might have translational implications.

## Nuclear IGF1R: A new layer of biological regulation

As alluded to above, the classical model of IGF1 action involves the ligand-induced phosphorylation of IGF1R, a heterotetrameric cell-surface tyrosine kinase receptor, with ensuing activation of cytoplasmic signaling cascades. The recent identification of nuclear IGF1R translocation provides an additional level of biological complexity by allowing a typical transmembrane receptor to function in a discrete, membrane-bound cellular environment (160–162). Using cell fractionation techniques and confocal microscopy it was shown that the cell-surface IGF1R undergoes modification by the small ubiquitin-like modifier protein (SUMO-1), with subsequent translocation to the nucleus (163, 164). SUMOylation sites on lysine residues within the tyrosine kinase domain are conserved among a variety of homologues from different species. Mutagenesis of these sites arrested nuclear import and gene activation (165).

Lysosomal and endocytic pathways that are mainly involved in IGF1R and INSR degradation were shown to be also responsible for nuclear translocation of the receptors (162). Importin-β, an important player in nuclear translocation, was shown to coimmunoprecipitate with IGF1R (164). In addition, use of the clathrin-dependent endocytosis inhibitor *dansylcadaverine* abrogated IGF1R nuclear import (162, 166). The capacity of IGF1R to interact with DNA was investigated by chromatin immunoprecipitation (ChIP)-seq assays. Analyses showed that the vast majority (~80%) of IGF1R-enriched regions were intergenic (*i.e*., distal from any annotated gene), while ~6% of these regions were located in introns and ~6% in exons (165). Hence, data is consistent with the notion that IGF1R may bind to enhancer regions and function as a transcriptional activator.

What are the clinical implications of nuclear IGF1R translocation? The impact of nuclear IGF1R on tumor aggressiveness can be deduced from the fact that inhibition of nuclear IGF1R import correlated with a reduced proliferative potential (167, 168). Aleksic and colleagues reported that nuclear IGF1R was present in renal cancer cells, preinvasive breast lesions and non-malignant tissues with a high proliferative index (162). Moreover, nuclear IGF1R staining correlated with an adverse prognosis in renal cancer. Similarly, nuclear IGF1R localization in alveolar rhabdomyosarcoma was associated with an aggressive phenotype. Finally, immunohistochemical analyses identified IGF1R staining in 47 out of 53 pediatric gliomas (169). Ten out of the 47 cases exhibited nuclear staining. IGF1R staining was mostly non-nuclear in low-grade tumors, while nuclear expression was predominant in high-grade gliomas. Survival was significantly longer in patients with gliomas having non-nuclear IGF1R localization than in patients with nuclear IGF1R. Taken together, data indicate that intracellular IGF1R distribution may help in stratifying pediatric glioma patients.

## IGF1R: An emerging therapeutic target in oncology

Following our analysis of cancer hallmarks from the perspective of the IGF1 signaling pathway, it is relevant to question what was the rationale behind the identification of IGF1R as a therapeutic target. Three main lines of research over the past 25 years led to the concept that the IGF1 axis and, in particular, the IGF1R is a potential goal for pharmacological (or other) intervention (1): during oncogenic transformation a “*primitive*” pattern of IGF1R expression is established, leading to enhanced IGF1R levels. A similar developmental trend is exhibited by IGF2, which is produced by most cancer cells and, usually, constitutes the main ligand in tumors (170, 171). These observations led to the *dogma* that IGF1R expression is a critical requirement for establishment of a tumor (15). This paradigm, however, is not necessarily true in *every* type of cancer. Thus, whereas IGF1R overexpression is a common trait of most pediatric and other solid tumors (*e.g*., brain, kidney), a more complex pattern of expression is seen in adult epithelial tumors (*e.g*., breast, prostate) (172–174); (2) further support to the notion that IGF1R might constitute a rational therapeutic target in oncology was provided by studies showing that cells deprived of the receptor, in their vast majority, do not undergo oncogenic transformation (81); and (3) the identification of endocrine IGF1 as a risk factor in multiple neoplasias generated the “*critical mass*” needed to proceed with clinical trials against the IGF1 axis (175). IGF1R-directed therapies are aimed at:

- inhibiting cancer cell survival and proliferation;
- reversing tumor growth and metastasis development; and
- sensitizing to chemotherapy, radiotherapy and biological therapies.

Different strategies have been developed to target the IGF system *in vitro* and in animal models (25, 27, 28, 176–178). However, three main strategies progressed to clinical trials (1): antibodies that target the IGF1R and induce its internalization and degradation; (2) small molecule IGF1R tyrosine kinase inhibitors; and (3) neutralizing antibodies that target the IGF1 and IGF2 ligands (179, 180). Unfortunately, most clinical trials led to disappointing results and it is nowadays clear that a combined approach aimed against the IGF axis along with additional pathway/s should lead to a better outcome (181). A recent pre-clinical study provided evidence that co-treatment of breast cancer cells with AEW541 (a selective IGF1R inhibitor) along with gemcitabine (a chemotherapeutic drug) improved the treatment efficiency (31). The degree of synergy achieved, as expressed in combination index values, was very strong. Finally, cell cycle analyses suggested that the synergism was derived, at least in part, from AEW541-induced G_1_ arrest and gemcitabine-induced S arrest.

Some of the IGF1R antibodies developed in recent years were shown to cross-react with the INSR leading to hyperglycemia. The potential effect of IGF1R antibodies on INSR signaling is of special concern given that these antibodies can alter INSR function, leading to insulin resistance and adverse effects on glucose and carbohydrate metabolism. On the other hand, INSR targeting could be potentially beneficial because inhibition of the INSR, in addition to IGF1R inhibition, might increase the effective anti-tumoral activity (182, 183). Hence, one of the critical challenges in the field is to define whether future trials should be limited to IGF1R or, alternatively, targeting tools should be implemented also against INSR.

## Concluding remarks: Future IGF research and the clinics

In summary, we believe that the negative outcomes of recent clinical trials, despite the obvious disappointment, were critically analyzed and important lessons were learned (26, 184, 185). Among other possible causes, a retrospective evaluation points out at a fierce competition between pharma companies as one of the reasons that hampered the development of efficient IGF1R drugs. These ‘battles’ led to the design of badly planned trials that, for the most part, were conducted on unselected patients. Therefore, it is of critical importance to identify predictive markers that could assist in selecting patients who might benefit from these treatments. Furthermore, biomarkers are also needed to monitor patient’s response to therapy. We believe that a rational and integrated use of *omics* platforms may certainly help identifying ‘IGF1 signatures’ that correlate with better clinical outcomes.

The question whether INSR is a druggable target in cancer is a matter of controversy. As mentioned in the previous section, a critical challenge is to define whether future trials should be limited to IGF1R or, alternatively, should target also INSR. Given the important role of insulin and INSR in cancer biology, mainly breast and endometrial tumors, it is expected that future efforts will embrace also the development of INSR-directed molecules.

As stated above, most available evidence indicates that selective IGF1R inhibitors along with chemotherapy or other biological drug (*i.e*., combination therapy) usually lead to a better outcome than monotherapy. The enhancement of the therapeutic effect stems from the fact that IGF1R therapy and chemotherapy are aimed against different phases or targets of the cellular machinery. Hence, whereas chemicals mostly induce DNA damage, IGF1R inhibitors specifically target the survival machinery of the cell.

Elucidation of the interplay between the IGF1 axis and additional pathways, including oncogenes and anti-oncogenes, will have a major impact on our understanding of basic molecular oncology processes as well as on our ability to design and optimize cancer therapies. A summary of the involvement of the IGF1 system in Cancer Hallmarks is presented in **Table 2**. The potential of new drugs, alone or in combination with chemotherapy or other biological agents, needs to be investigated in randomized studies. Finally, lessons from the field of personalized medicine will be implemented in IGF1R targeting.

**Table 2:** Hallmarks of cancer and the IGF1 signaling pathway.

CANCER HALLMARK	IGF1 INVOLVEMENT
**Sustained proliferative signaling**	IGF1 stimulates proliferation and inhibits death in a variety of cell types. IGF1 functions as a progression factor required to traverse the cell cycle. IGF1 has an important role in stem cell biology.
**Insensitivity to antigrowth signals**	Cancer genes (*e.g*., tumor suppressors, oncogenes) adopt the IGF1 signaling pathway. Cellular and viral oncogenes require an intact IGF1 axis in order to elicit their transforming roles.
**Evasion of apoptosis**	The IGF1R exhibits a potent anti-apoptotic activity. IGF1R confers upon cells enhanced survivability. Cells deprived of the IGF1R do not undergo transformation.
**Genome instability and mutation**	p53, a DNA integrity sensor, suppresses *IGF1R* gene transcription. Mutation of p53 enhances *IGF1R* gene expression and IGF1-mediated mitogenesis. Activation of BRCA1 upon DNA damage leads to a reduction in IGF1R levels and IGF1 action.
**Sustained angiogenesis**	IGF1 enhances the hypoxic response by stabilizing HIF-α and upregulating VEGF-A. A functional cooperation between the IGF1R and VEGF pathways has been identified in cancer cells.
**Tissue invasion and metastasis**	Deregulated expression and availability of IGFs are regarded as key regulators of metastasis. Obesity leads to changes in the extracellular matrix and adipose cells, creating a cancer-permissive microenvironment.

## Author contributions

HW and DL: substantial contributions to the conception and design of the work and interpretation of data for the work; drafting the work and revising it critically for important intellectual content; final approval of the version to be published; and agreement to be accountable for all aspects of the work in ensuring that questions related to the accuracy or integrity of any part of the work are appropriately investigated and resolved. Both authors contributed to the article and approved the submitted version.

## Funding

Work in the laboratory of HW is supported by grants from the Israel Science Foundation, US-Israel Binational Science Foundation, the Israel Cancer Association and the Recanati Foundation (Tel Aviv University). Work in the laboratory of DL is supported by NCI grant R01CA128799.

## Acknowledgments

HW is the incumbent of the Lady Davis Chair in Biochemistry.

## Conflict of interest

The authors declare that the research was conducted in the absence of any commercial or financial relationships that could be construed as a potential conflict of interest.

## Publisher’s note

All claims expressed in this article are solely those of the authors and do not necessarily represent those of their affiliated organizations, or those of the publisher, the editors and the reviewers. Any product that may be evaluated in this article, or claim that may be made by its manufacturer, is not guaranteed or endorsed by the publisher.
